# Kinase Screening in *Pichia pastoris* Identified Promising Targets Involved in Cell Growth and *Alcohol Oxidase 1* Promoter (P*_AOX1_*) Regulation

**DOI:** 10.1371/journal.pone.0167766

**Published:** 2016-12-09

**Authors:** Wei Shen, Chuixing Kong, Ying Xue, Yiqi Liu, Menghao Cai, Yuanxing Zhang, Tianyi Jiang, Xiangshan Zhou, Mian Zhou

**Affiliations:** 1 State Key Laboratory of Bioreactor Engineering, East China University of Science and Technology, Shanghai, China; 2 Shanghai Collaborative Innovation Center for Biomanufacturing (SCICB), Shanghai, China; 3 Roche R&D Center (China) Ltd, Pudong, Shanghai, China; National Renewable Energy Laboratory, UNITED STATES

## Abstract

As one of the most commonly used eukaryotic recombinant protein expression systems, *P*. *pastoris* relies heavily on the *AOX1* promoter (P_*AOX1*_), which is strongly induced by methanol but strictly repressed by glycerol and glucose. However, the complicated signaling pathways involved in P_*AOX1*_ regulation when supplemented with different carbon sources are poorly understood. Here we constructed a kinase deletion library in *P*. *pastoris* and identified 27 mutants which showed peculiar phenotypes in cell growth or P_*AOX1*_ regulation. We analyzed both annotations and possible functions of these 27 targets, and then focused on the MAP kinase Hog1. In order to locate its potential downstream components, we performed the phosphoproteome analysis on glycerol cultured WT and Δ*hog1* strains and identified 157 differentially phosphorylated proteins. Our results identified important kinases involved in *P*. *pastoris* cell growth and P_*AOX1*_ regulation, which could serve as valuable targets for further mechanistic studies.

## Introduction

As one of the most commonly used expression systems, *P*. *pastoris* is highly efficient and cost effective for both secretive and intracellular protein expression. Several important features of *P*. *pastoris* render it ideally suitable for large-scale production of recombinant proteins [1]. So far, over 5000 recombinant proteins have been successfully expressed in *P*. *pastoris* including insulin, α-interferon and hepatitis B antigen (http://www.pichia.com/). In most cases, recombinant protein expression in *P*. *pastoris* relies on the *AOX1* gene promoter (P_*AOX1*_). *AOX1* is the major gene encoding alcohol oxidase (Aox), which is substantially induced when cells are cultured in methanol and occupies 30% of total soluble proteins in the yeast cell [2]. *P*. *pastoris* belongs to the group of methylotrophic yeasts which are capable of utilizing methanol as the sole carbon and energy source for cell growth. While strongly induced by methanol, P_*AOX1*_ is strictly repressed by other carbon sources such as glucose, glycerol and ethanol [3].

P_*AOX1*_ is not directly activated or repressed by carbon containing nutrients, but rather regulated by complicated cell signaling pathways that remain to be elucidated. So far several protein factors have been reported to participate in P_*AOX1*_ regulation. Hexose sensor Gss1 [4] and transporter Hxt1 [5] were reported to function at the first stage of P_*AOX1*_ repression pathways when *P*. *pastoris* are cultured in glucose. Deficiency of either of the genes led to the de-repression of P_*AOX1*_ in response to glucose. In addition, some downstream transcriptional repressors such as Nrg1 [6] and activators (Mit1, Prm1 and Mxr1) have been identified to regulate P_*AOX1*_ [7,8].

Kinases are well-known to play important roles in cell signaling, since phosphorylation and de-phosphorylation processes are crucial for the on and off of a wide variety of biological activities. For example, phosphorylation is crucial for the cellular localization and activity of ScMig1 (*S*. *cerevisiae* Mig1), which functions in glucose mediated gene repression [9,10]. Another example lies in *H*. *polymorpha* glucokinase and hexokinase. In the hexokinase knock out mutant, glucose or fructose failed to repress the alcohol oxidase gene promoter [11–13]. Parua et al have shown that Ser215 phosphorylation is necessary for the interaction between 14-3-3 proteins and P_*AOX1*_ positive regulator Mxr1 in *Pichia pastoris* [14]. However, few kinases have been identified to be involved in P_*AOX1*_ activation/repression in *P*. *pastoris* so far. To address that, we performed a kinase screening by knocking out the predicted kinases one by one and examined the cell growth rates and alcohol oxidase activities on different carbon sources. As a result, we identified a few kinase mutants which showed peculiar phenotypes in cell growth or P_*AOX1*_ regulation. Then we focused on the MAP kinase Hog1 and performed a phosphoproteome analysis on WT and Δ*hog1* strain to locate any possible Hog1 downstream components.

## Results

**Fig 1:**
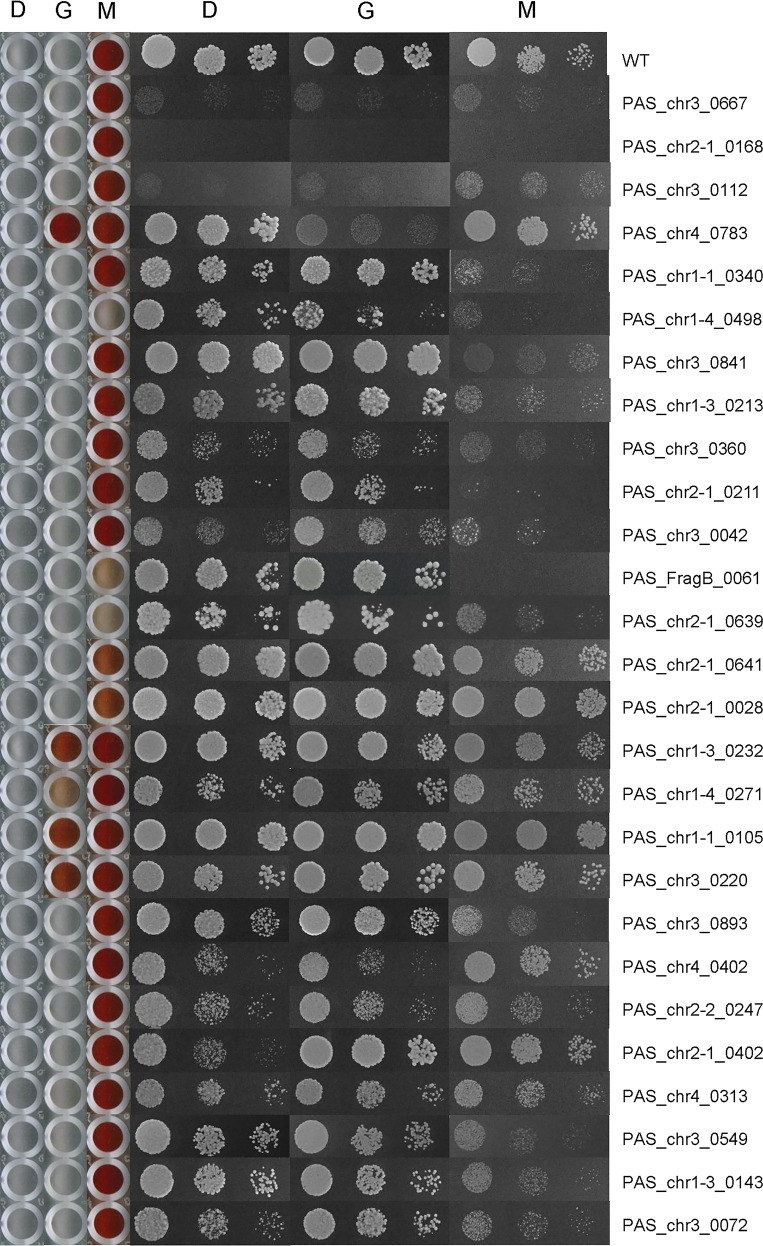
The growth rates (shown by spotting assay) and Aox activities (shown by colorimetrical assay) of the 27 knockouts. D: glucose; G: glycerol; M: methanol.

**Fig 2:**
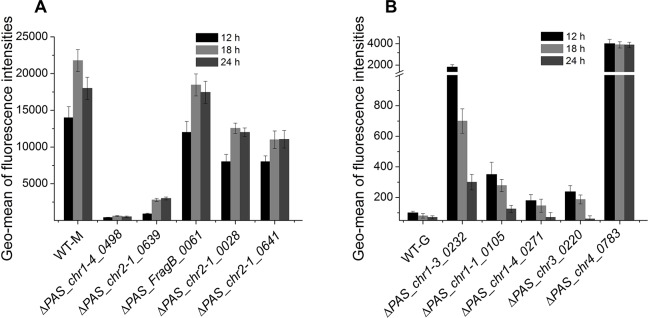
GFP reporter assay showing the AOX1 promoter activity of several kinase mutants in methanol (A) or glycerol (B). A WT strain is used as control here.

Of the total of 152 annotated kinases in the whole genome of *P*. *pastoris*, we have successfully knocked out 92 of them. The failure to knock out the rest of them may be due to the lethality caused by losing an essential kinase. Details on kinase screening methods are shown in Materials and methods part. After examining cell growth and P_*AOX1*_ activity of these knockout strains on glucose, glycerol or methanol, we identified 27 kinases involved in cell growth or P_*AOX1*_ regulation. (Spotting assay and OD measurement in liquid culture were combined to test cell growth rate. Enzymatic activity of Aox was measured to represent *AOX1* promoter activity. However, in order to exclude any possibilities of post-transcriptional and post-translational control, a P_*AOX1*_-GFP reporter was expressed in those mutants with interesting phenotypes to confirm the promoter activity.) The growth rates and Aox enzymatic activities of all of the 92 knockout strains on three carbon sources are shown in Fig A in S1 File. The phenotypes of the 27 affected kinase knockouts were shown in Fig 1, Fig 2, Fig B in S1 File and summarized in Table 1.

**Table 1:** Summary of cell growth and Aox enzymatic activities of the 27 kinase genes.

Gene	Growth (glucose)	Growth (glycerol)		Growth (methanol)		Aox activity (glucose)		Aox activity (glycerol)		Aox activity (methanol)
*PAS_chr3_0667*	−−−		−−−		−−−					
*PAS_chr2-1_0168*	−−−		−−−		−−−					
*PAS_chr3_0112*	−−−		−−−		−−					
*PAS_chr4_0783*			−−−						+++	
*PAS_chr1-1_0340*	−		−		−−−					
*PAS_chr1-4_0498*	−		−−		−−−					—
*PAS_chr3_0841*					−−−					
*PAS_chr1-3_0213*	−		−		−−−					
*PAS_chr3_0360*	−		−		−−−					
*PAS_chr2-1_0211*	−		−		−−−					
*PAS_chr3_0042*	−		−		−−−					
*PAS_FragB_0061*					−−−					—
*PAS_chr2-1_0639*	−		−		−−					—
*PAS_chr2-1_0641*										-
*PAS_chr2-1_0028*										-
*PAS_chr1-3_0232*									+++	
*PAS_chr1-4_0271*	−		−						++	
*PAS_chr1-1_0105*									++	
*PAS_chr3_0220*	−		−						+++	
*PAS_chr3_0893*					−					
*PAS_chr4_0402*	−		−							
*PAS_chr2-2_0247*	−		−		−					
*PAS_chr2-1_0402*	−									
*PAS_chr4_0313*	−		−		−					
*PAS_chr3_0549*					−−					
*PAS_chr1-3_0143*					−−					
*PAS_chr3_0072*	−				−−					

Growth defect or decreased Aox enzymatic activity are marked by “−”, whereas elevated Aox enzymatic activity is marked by “+”. “+” or “-” represents mild effect, “++”or “−-” represents moderate while “+++” or “---” represent severe effect.

### Kinases involved in cell growth on different carbon sources

Among the 92 knockouts, a total of 23 strains showed growth defects on one or more carbon sources (Fig 1, Fig B in S1 File and Table 1). Among them, Δ*PAS_chr3_0667*, Δ*PAS_chr2-1_0168* and Δ*PAS_chr3_0112* showed most severe phenotypes, *i*.*e*. growth arrest on all the three carbon sources. *PAS_chr3_0667* is annotated as adenylylsulfate kinase, required for sulfate assimilation and involved in methionine metabolism. Its homologous gene in *S*. *cerevisiae* is called *MET14* and is involved in methionine biosynthesis [15]. *PAS_chr2-1_0168* gene product is processed in mitochondria to yield acetylglutamate kinase and N-acetyl-gamma-glutamyl-phosphate reductase. Its homolog in *S*. *cerevisiae* is *ARG5*,*6*, which acts at the second and third steps of arginine biosynthesis in mitochondria [16]. This indicates that both of the genes may play a key role in amino acid biosynthesis in *P*. *pastoris*. *PAS_chr3_0112* is annotated as an essential serine kinase involved in cell cycle progression. Therefore these three kinases could be considered as general but not carbon source specific factors involved in the regulation of cell growth.

Δ*PAS_chr4_0783* specifically showed severe growth defect on glycerol but not on the other two types of carbon sources. This gene encodes a glycerol kinase that converts glycerol to glycerol-3-phosphate. Its homolog in *S*. *cerevisiae* functions at the first step of glycerol usage through phosphorylation [17]. In addition, the fact that Δ*PAS_chr4_0783* showed extremely high Aox enzymatic activity (Fig 1) and promoter activity (Fig 2B) on glycerol indicates the possibility that this kinase is involved in both glycerol utilization and glycerol mediated P_*AOX1*_ repression.

Besides, cell growth of Δ*PAS_chr1-4_0340*, Δ*PAS_chr1-4_0498*, Δ*PAS_chr3_0841*, Δ*PAS_chr1-3_0213*, Δ*PAS_chr3_0360*, Δ*PAS_chr2-1_0211*, Δ*PAS_chr3_0042 and* Δ*PAS_FragB_0061* was largely inhibited on methanol. *PAS_chr1-4_0498* is annotated as one of three possible beta-subunits of the Snf1 kinase complex. In *S*. *cerevisiae*, Snf1 is one of the key regulators of carbohydrate metabolism and the kinase deletion mutant fails to grow on non-fermentable carbon source [18]. Taken together with the fact that Δ*PAS_chr1-4_0498* showed extremely low Aox enzymatic activity on methanol, it is likely that PpSnf1 is involved in both methanol utilization and methanol mediated P_*AOX1*_ activation. *PAS_chr3_0841* is annotated as dihydroxyacetone kinase (*DAK*) which converts dihydroxyacetone (DHA) to dihydroxyacetone phosphate (DHAP). The annotation and homologs of the kinase genes mentioned above are summarized in Table 2. Additional investigations are needed to discover the detailed functions of these kinases.

**Table 2:** Summary of kinase genes specifically involved in methanol supported cell growth.

Kinase gene	Annotation	*S*. *cerevisiae* homolog	Reference
*PAS_chr1-4_0340*	Protein kinase involved in bud growth and assembly of the septin ring	*GIN4*, involved in bud growth	[19]
*PAS_chr1-4_0498*	One of three possible beta-subunits of the Snf1 kinase complex	*GAL83*, key regulator of carbohydrate metabolism	[18]
*PAS_chr3_0841*	Dihydroxyacetone kinase (*DAK*)	*DAK2*, involved in detoxification of dihydroxyaectone	[20]
*PAS_chr1-3_0213*	Protein kinase	n/a	
*PAS_chr3_0360*	Mitochondria protein kinase	*PKP2*	
*PAS_chr2-1_0211*	Subunit of phosphoatidylinositol (PtdIns) 3-kinase complex I and II	VPS30, involved in vacuolar protein sorting and often functioned in autophagy	[21]
*PAS_chr3_0042*	Myristoylated serine/threonine protein kinase	VPS15, involved in vacuolar protein sorting and often functioned in autophagy	[21]
*PAS_FragB_0061*	Phosphoenolpyruvate carboxykinase	*PCK1*, converts oxaloacetate into phosphoenolpyruvate	[22]

### Kinases involved in P_*AOX1*_ regulation

P_*AOX1*_ is normally activated by methanol but repressed by glucose and glycerol. During our screening, we identified several knockouts with unexpected P_*AOX1*_ activities. As shown in Fig 1 and Table 1, methanol induced Aox enzymatic activities in Δ*PAS_chr2-1_0641* and Δ*PAS_chr2-1_0028* strains were mildly affected, but were largely diminished in Δ*PAS_chr2-1_0639*, Δ*PAS_chr1-4_0498* and Δ*PAS_FragB_0061*. By checking GFP fluorescence after expressing it under P_*AOX1*_ (Fig 2A), Δ*PAS_chr2-1_0639* and Δ*PAS_chr1-4_0498* has the most severely reduced P_*AOX1*_ activity in methanol. As mentioned previously, Δ*PAS_chr1-4_0498* encodes one of the three possible beta-subunits of the Snf1 kinase complex, while *PAS_chr2-1_0639* is annotated as upstream serine/threonine kinase for the *SNF1* complex. This indicates that PpSnf1 is an important kinase mediating P_*AOX1*_ activation by methanol.

Similarly, five knockouts showed strong or moderate Aox enzymatic activities on glycerol, which indicates the possible roles of the corresponding kinases, *i*.*e*. *PAS_chr1-3_0232*, *PAS_chr1-4_0271*, *PAS_chr1-1_0105*, *PAS_chr3_0220* and *PAS_chr4_0783*, in glycerol mediated P_*AOX1*_ repression. P_*AOX1*_-GFP fluorescence suggested that all of them have elevated P_*AOX1*_ activity with different levels (Fig 2B). By checking their annotations and *S*. *cerevisiae* homologs, the first four genes attracted our attention as they all belong to the MAPK signaling pathway. *PAS_chr1-3_0232* is a mitogen-activated protein kinase (MAP Kinase, MAPK); *PAS_chr1-4_0271* and *PAS_chr1-1_0105* are annotated as a MAP kinase kinase (MAPKK);and *PAS_chr3_0220* is annotated as MAP kinase kinase kinase (MAPKKK). Their *S*. *cerevisieae* homologs are *HOG1*, *MKK1*, *PBS2* and *BCK1*, respectively. In the osmosensing pathway, ScHog1 is a direct substrate of ScPbs2 [23], whereas ScMkk1 is a direct substrate of ScBck1 in cell integrity pathway [24]. Based on the screening results here, it is possible that these four kinases may work together to mediate P_*AOX1*_ repression by glycerol. *PAS_chr1-3_0232* is then named Pp*HOG1*.

### Phosphoproteome analysis in WT and Δ*hog1* strains

**Fig 3:**
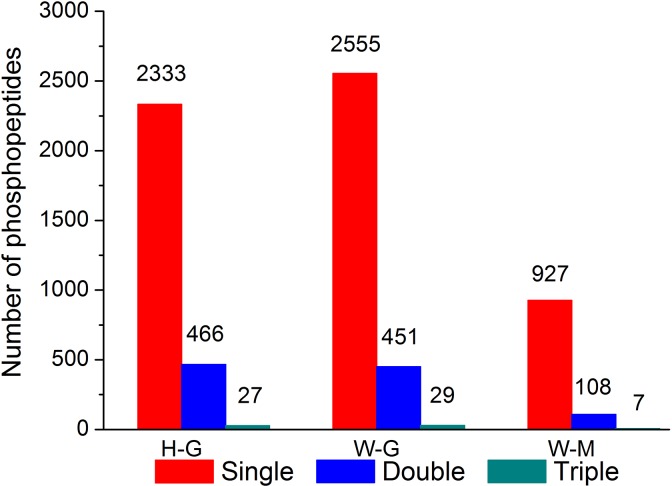
The counts of phosphopeptides carrying single, double and triple phosphate groups in H-G, W-G and W-M. H-G: glycerol cultured Δ*hog1* strain; W-G: glycerol cultured WT strain; W-M: methanol cultured WT strain.

In order to identify the downstream components of PpHog1, a phosphoproteome analysis was conducted to compare the phosphoprotein profiles in methanol cultured WT strain (W-M) as well as glycerol cultured WT (W-G) strain and Δ*hog1* strain (H-G). For each sample, phosphopeptides were enriched by TiO_2_ columnand analyzed by LC-MS/MS assay. A total of 2826, 3035 and 1042 phosphopeptides were identified covering 2738, 2966 and 1024 phosphosites in H-G, W-G and W-M samples respectively (Table 3). For all the three samples, most phosphopeptides carried only one phosphate group, and around 10–17% phosphopeptides carried two phosphorylation sites. Modifications with three or more phosphate groups were rarely detected (Fig 3). The numbers of phosphoserine, phosphothreonine and phosphotyrosine in each sample are listed in Table 3. By aligning these phosphopeptides to the database, we finally detected 958, 1016 and 498 phosphoproteins in H-G, W-G and W-M, respectively.

**Table 3:** The characteristics of the identified phosphoproteins.

Sample	Phospho-sites	Phospho_Ser	Phospho_Thr	Phospho_Tyr	Phospho-peptides	Phospho-proteins
H-G	2738	2255	463	20	2826	958
W-G	2966	2441	500	25	3035	1016
W-M	1024	855	160	9	1042	498

### Gene ontology (GO) analysis of phosphoproteome

**Fig 4:**
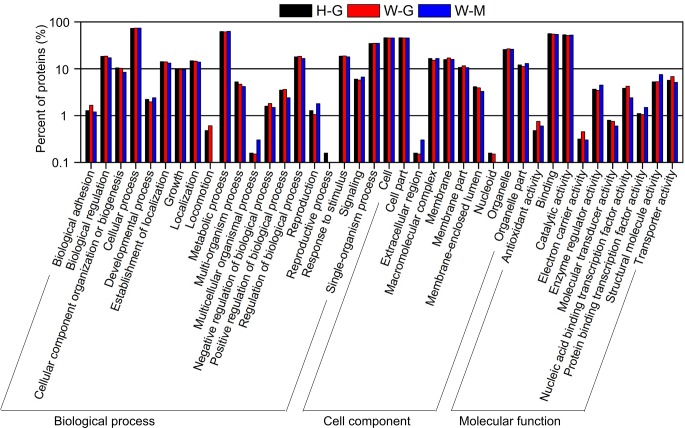
A gene ontology analysis of the phosphoproteins in H-G, W-G and W-M. H-G: glycerol cultured Δ*hog1* strain; W-G: glycerol cultured WT strain; W-M: methanol cultured WT strain.

A gene ontology analysis was conducted in the vocabulary of “biological process”, “cellular component” and “molecular function”, and each category could be further classified into a few subcategories. The distributions of H-G and W-G were quite similar, except in “reproductive process”. The W-M profile differed from other two strains in “locomotion”, “multicellular organismal process”, “reproductive process”, “extracellular region” and “nucleoid” (Fig 4).

### Cluster of Orthologous Groups (COG) analysis of phosphoproteome

**Fig 5:**
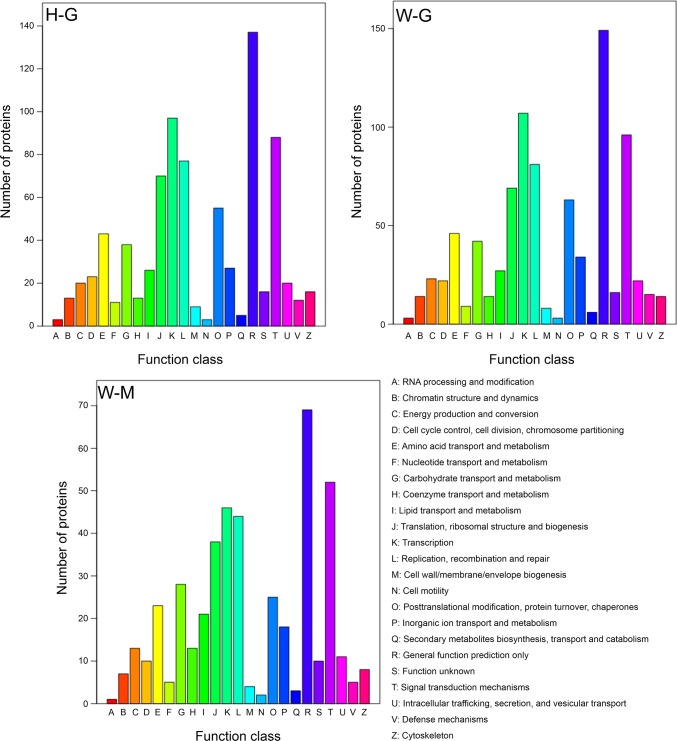
A cluster of orthologous groups of proteins analysis of the phosphoproteins in H-G, W-G and W-M. H-G: glycerol cultured Δ*hog1* strain; W-G: glycerol cultured WT strain; W-M: methanol cultured WT strain.

COG is a database classified according to protein’s homology. We blasted the identified phosphoproteins in COG database and classified them into corresponding groups. Despite some minor differences, the COG profiles of phophoproteins in H-G, W-M and W-G cells were quite similar (Fig 5).

### The identified possible substrates of PpHog1

**Fig 6:**
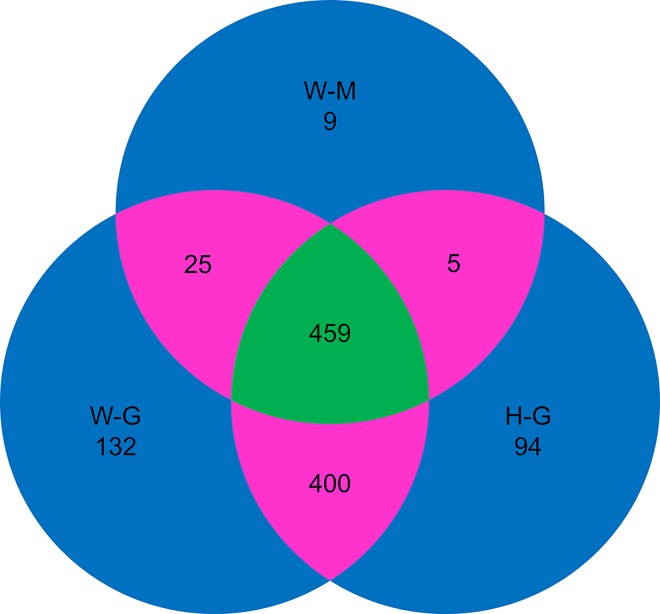
A venn diagram showing the number of phosphoproteins identified in H-G, W-G and W-M. Blue shaded area marks the number of phosphoproteins specifically identified in one strain. Pink shaded area makes shared phosphoproteins in two strains, while green area labels the common phosphoproteins identified in all three strains. H-G: glycerol cultured Δ*hog1* strain; W-G: glycerol cultured WT strain; W-M: methanol cultured WT strain.

In H-G, W-G and W-M strains, a total of 1124 phosphoproteins were obtained. A complete list could be found in S2 Table. As summarized in Fig 6, among these phosphoproteins 94, 132 and 9 were specifically phosphorylated in H-G, W-G and W-M, respectively (shaded by blue). Some phosphoproteins were shared by two of these strains (shaded by pink), and 459 proteins were found to be phosphorylated in all samples (shaded by green). Generally the proteins in methanol cultured strain were much less phosphorylated than glycerol cultured strains. The 157 (132+25) proteins specifically phosphorylated in W-G but not in H-G are potential direct or indirect targets of PpHog1. The annotation description and homolog analysis of their coding genes with reference to three databases (NCBI, SwissProt and TrEMBL) are listed in S1 Table.

## Discussion

In this paper, we designed a kinase screening assay in *P*. *pastoris* and identified 27 targets involved in cell growth or P_*AOX1*_ regulation. Our results demonstrated that (1) 23 kinases are involved in regulating cell growth on different carbon sources and to different extents. (2) 5 kinases are involved in P_*AOX1*_ activation and 5 kinases are involved in P_*AOX1*_ repression, most of which are related to Snf1 and Hog1 Pathways. (3) The phosphoproteome analysis revealed possible downstream components involved in *PAS_chr1-3_0232* (Pp*HOG1*) mediated P_*AOX1*_ repression under glycerol. We summarized and plotted the possible roles of these mutations in regulating P_*AOX1*_ (Fig D in S1 File). Future investigations are needed to examine the roles and functions of these targets.

Mizuno *et al*. and Piao *et al*. reported recently that ScSnf1 could modulate the activity of ScHog1 through its upstream kinase ScSsk1, which is independent of the classical osmosensing pathway [25,26]. In our screening, we identified PpSnf1 subunit PpGal83 (*PAS_chr1-4_0498*) and PpHog1 (*PAS_chr1-3_0232*) involved in positive and negative regulation of P_*AOX1*_ respectively. It is likely that these two components have crosstalk in the P_*AOX1*_ regulation process. Further studies are needed to reveal the detailed mechanism.

Cell growth defect is not necessarily linked with affected Aox enzymatic activity. For example, in Δ*PAS_chr3_0667*, Δ*PAS_chr2-1_0168 and* Δ*PAS_chr2-1_0211* (Fig 1), cell growth in methanol were completely abolished but Aox enzymatic activity was not affected. Since Aox enzymatic activity is not a direct sign to represent P_*AOX1*_ activity, we checked P_*AOX1*_ activity more directly by putting a GFP reporter gene under it (Fig 2). We also checked Aox protein and mRNA levels in some strains with reduced enzymatic activity in methanol (Fig C in S1 File). For those mutants in which promoter activity matches well with protein level and enzymatic activity, the corresponding kinases are likely to be involved in P_*AOX1*_ regulation. Otherwise, the kinase may be partially redundant for P_*AOX1*_ regulation, or participate in the post-translational control of Aox to regulate its activity.

Besides *P*. *pastoris*, *H*. *polymorpha*, *C*. *boidinii* and *P*. *methanolica* are the most typical examples of methalotrophic yeasts [1,27]. In order to metabolize methanol, they all express a conserved alcohol oxidase likely with similar regulation process Therefore, our results may also contribute to the understanding of the important components for alcohol oxidase gene regulation in other methalotrophic yeast species.

## Materials and Methods

### Strains and culture conditions

*P*. *pastoris* GS115 (Invitrogen) was used as the wild type (WT) strain. Unless indicated otherwise, *P*. *pastoris* strains were grown at 30°C in YPD medium [1% (w/v) yeast extract, 2% (w/v) peptone, 2% (w/v) glucose] or minimal YNB medium [0.67% (w/v) yeast nitrogen base with histidine] supplemented with different carbon sources, *e*.*g*., 1% (w/v) glucose (YND), 1% (w/v) glycerol (YNG), or 0.5% (v/v) methanol (YNM). For solid media, agar was added 2% (w/v). Cell density (OD_600_) was determined spectophotometrically at the wavelength 600 nm. *E*. *coli* Top10 cells were used for plasmid propagation.

### Construction of the *P*. *pastoris* kinase mutant library

We referred to the *P*. *pastoris* GS115 genome data base (http://www.ncbi.nlm.nih.gov/genome/?term=GS115) and found 152 annotated kinases. For each kinase, the knockout strain was constructed by the gene replacement method using the Zeocin resistance gene *Sh ble* as a marker. First of all, the upstream region, Zeocin resistance gene *Sh ble* and the downstream region were amplified by PCR. Primers were designed according to Vazyme ClonExpress MultiS One Step Cloning Kit (Weizan Ltd., Shanghai, China). Secondly, the three fragments were ligated into the pUC18 vector digested with *Bam*HI/*Pst*I to yield a kinase deletion vector. The deletion cassette was then amplified and transformed into *P*. *pastoris* GS115 strain by electroporation. After cultured on YPD (supplement 100 μg/mL Zeocin) for 2–4 days, the strain with correct replacement of the original kinase gene with deletion cassette was picked and confirmed by PCR.

### Cell growth and Aox enzymatic activity assays

The knockout strains were pre-grown in YPD media to OD_600_ of 2–8. Then the cells were harvested by centrifugation at 3000 *g* for 5 minutes and washed three times with sterile water. To examine growth, cell suspensions were spotted 5 μL in three dilutions (0.1, 0.01 and 0.001 OD_600_) onto YND, YNG and YNM medium, respectively. The strains were grown for 2 days on YND and YNG while 3 days on YNM.

The colorimetrical assay was used to measure Aox enzymatic activity. Cells were re-suspended in 500 μL YNB supplemented with different carbon sources in 96 deep well plates to reach OD_600_ of 1.0. After 6 hours culture on a shaker (200 rpm, 30°C), 1 mL reaction buffer was added. After incubation for 20 min, 100 μL mixer was transferred into another 96-well plate and scanned. The reaction buffer for colorimetrical assay including 0.05% (w/v) O-dianisidine, 0.15% (w/v) CTAB, 1% (v/v) methanol, 3 U/mL HRP, and 100 mmol/L potassium phosphate buffer (pH 7.0).

### Cell growth in liquid culture

The strains were pre-grown in YPD media to OD_600_ of 2–8. The cells were harvested by centrifugation at 3,000 *g* for 5 minutes, washed three times with sterile water, and re-suspended with initial OD_600_≈1.0 in 50 mL YNB media supplemented with various carbon sources. At suitable intervals, OD_600_ was measured for growth curve.

### GFP reporter assay

The GFP reporter gene was constructed after P_*AOX1*_, and transformed into the kinase mutants. For measuring GFP, 1 mL aliquot of culture media was removed, and cells were harvested by centrifugation and then stored at -80°C. when measuring, frozen cells were thawed, washed twice with sterile water, and transferred into 96-well plates with diluting to about OD_600_≈1. OD_600_ and GFP were measured by enzyme-labeled instrument (BioTek) with three biological replicates.

### Cell extract preparation and Western blot analysis

To prepare cell extracts, 30–50 OD_600_ units of cells were harvested by centrifugation at 6,000 *g* for 3 min, washed twice with ice-cold 50 mM potassium phosphate buffer (pH 7.0), and then frozen at -20°C. Cells were thawed and re-suspended in 1 mL lysis buffer [50 mM potassium phosphate buffer (pH 7.0), 1 mM phenylmethylsulfonyl fluoride (PMSF)]. Aliquots of 1 mL were mixed with 1.8 g glass beads (Biospec Products, Bartlesville, OK) in a 2.0 mL screw-cap tube followed by disruption with a bead disrupter (Mini-BeadBeater-8; Biospec Products) for 8 cycles (1 min vibrating and 1 min resting in ice for each cycle). The lysate was centrifuged at 20,000 *g* for 30 min, the pellet was discarded, and the supernatant was utilized for Western blot. The protein concentration was determined with a Bradford protein assay kit (Tiangen, Shanghai, China).

Each lane was loaded 10 μg total proteins for SDS-PAGE and then transferred onto a polyvinylidene difluoride (PVDF) membrane using the electrophoretic transfer method with rabbit anti-Aox antibody (a kind gift from Suresh Subramani, University of California, San Diego) as the primary antibody and peroxidase-conjugated goat anti-rabbit immunoglobulin G as the secondary antibody.

### Phosphoproteome assay

The phosphoproteome assay was carried out by the Beijing Genomics Institute. The WT GS115 and the Δ*hog1* strain were pre-grown in YPD media until OD_600_ reached 2–8. Cells were harvested by centrifugation at 3000 *g* for 5 minutes and washed three times with sterile water. The cells were re-suspended in 50 mL medium supplemented with indicated carbon source with initial OD_600_ = 1.0. After incubation for 6 hours, cells were harvested by centrifugation at 3000 *g* for 5 minutes and frozen at -80°C. Then the cells were send to Beijing Genomics Institute for phosphoproteome assay.

As the protocol provided by Beijing Genomic Institute, phosphoproteome assay was performed in the following steps:

A. Total protein extraction: (1) 500 μL lysis buffer was added to dissolve cell pellets. Then the reaction was supplemented with PMSF (final concentration 1 mM), EDTA (final concentration 2 mM) and DTT (final concentration 10 mM, added after 5 min ice bath); (2) Cells were broken by sonication for 15 min, and centrifuged for 20 min (25000 *g*) to get the supernatant; (3) 5 times volume of cold acetone was added to the supernatant, and the mixer was incubated at -20°C for 2 hours. Then supernatant was removed by centrifugation (20 min, at 16000 *g*); (4) Repeat the step (1) and (2), and get the supernatant; (5) Treat the supernatant with DTT (final concentration 10 mM) at 56°C for 1 hour to open the disulfide bond. (6) Add IAM (final concentration 55 mM) and incubate in dark for 45 min to alkylate; (7) Appropriate amount of cold acetone was added again to precipitate proteins (incubated at -20°C, 2 hours). Supernatant was trashed by centrifugation (20 min, 25000 *g*); (8) Dissolve the pellet in 200 μL 0.5M TEAB with sonication; (9) Collect the supernatant after centrifugation (20 min, 25000 *g*) for quantification.

B. Enzymatic digestion of proteins: (1) Take 1000 μg proteins from each sample; (2) Trypsin (Protein to enzyme ration 20:1) was added to each sample and incubated at 37°C for 4 hours; (3) Trypsin of the same ratio was added one more time and to digest for another 8 hours at 37°C.

C. Phosphopeptide enrichment: (1) Peptides after trypsin digestion were desalted using Strata X C18 column and then vacuum dried; (2) 1000 μg dry peptides were re-dissolved with the binding buffer (65% ACN, 2% TFA), and saturated with glutamate (20 mg/mL, pH 2.0–3.5); (3) Then the solution was incubated with 500 μg balanced TiO_2_ column (GL Science, Saitama, Japan) to 20 min to enrich phosphopeptides; (4) The column was washed by wash buffer 1 (65% ACN, 0.5% TFA PH 2–3.5) and wash buffer 2 (65% ACN, 0.1% TFA PH 2–3.5); (5) The phosphopeptides were then eluted by elution buffer 1 (50% CAN, 1.1% NH_4_OH) and elution buffer 2 (50% CAN, 3% NH_4_OH); (6) The products were subsequently vacuum dried for LC-MS/MS analysis.

D. LC-ESI-MS/MS: Each dried fraction was dissolved in buffer A (5% ACN, 0.1% TFA) to the final concentration of 0.5 μg/μL, and centrifuged at 20000 g to get rid of any insoluble matter. Prominence nano (LC-20AD by SHIMADZU) was used for separation. For each fraction, 5 μL (2.5 μg proteins) was loaded onto the Trap column at a flow rate of 8 μL/min in4 min. Then an analyzing gradient with total flow rate 300 nL/min brought the samples to the analyzing column, separating samples and sending them to the LC-MS/MS system. Elution was carried out under 5% buffer B (95% ACN/0.1% TFA) for 5 min, followed by a 35 min-long liner gradient of buffer B from 5% to 35%. Afterwards, buffer B concentration was increased to 60% for 5 min, then to 80% in 2 min and held for 2 min. Finally, buffer B concentration decreased to 5% and the column was equilibrated for 10 min under this condition. The phosphopeptides were analyzed by Triple TOF 5600 (AB SCIEX). Raw spectral data was obtained from UniProt *Komagataella pastoris* (strain GS115) 5073 seqs (http://www.uniprot.org/uniprot/?query=taxonomy%3a644223&format=*). The peptide mass tolerance was set to ±0.05 Da and the fragment mass tolerance was set to ±0.1 Da. Gln->pyro-Glu (N-term Q), Oxidation (M) and Phospho (S/T/Y) was set as variable modifications, Carbamidomethyl (C) as fixed modifications, and two missed cleavage was allowed. The candidate phosphopeptides were initially assigned by ESI-MS/MS using 79.96 Da mass increments per phosphate moiety relative to the unmodified peptides. To detect the phosphopeptides, we utilized the preferred loss of the phosphate group upon collision-induced dissociation. In positive ion tandem MS, an intense neutral loss of 98 Da, corresponding to H_3_PO_4_, was observed for peptides containing phosphorylated Ser, Thr and Tyr residues.

## Supporting Information

S1 File Fig A: The growth rates (shown by spotting assay) and Aox enzymatic activities (shown by colorimetrical assay) of the 92 knockouts. Fig B: The growth rates (shown by OD measurement of liquid culture) of the 27 affected knockouts. D: glucose; G: glycerol; M: methanol. Fig C: The Aox protein and mRNA levels of three knockouts (Δ*PAS_1–4_0498*, Δ*PAS_FragB_0061* and Δ*PAS_chr2-1_0639*) cultured in methanol. Methanol and glycerol cultured WT strains (WT-M and WT-G) serve as positive and negative controls, respectively. Actin is blotted as a loading control. Fig D: Putative regulatory pathway of *AOX1* promoter in *P*. *pastoris*. The regulatory profiles of *AOX1* promoter in glycerol and methanol are described.
(PDF)

S1 Table The annotation description and homolog analysis against three database (NCBI, SwissProt and TrEMBL) of potential direct or indirect targets of PpHog1. (XLSX)

S2 Table A list of 1124 phosphoproteins identified in Fig 6. (XLSX)
