# Supplementary figures and images for: Kinase Screening in Pichia pastoris Identified Promising Targets Involved in Cell Growth and Alcohol Oxidase 1 Promoter (PAOX1) Regulation
Source: PLoS One. 2016 Dec 9;11(12):e0167766. doi: 10.1371/journal.pone.0167766 (PMC5147967; doi:10.1371/journal.pone.0167766)

Fig A

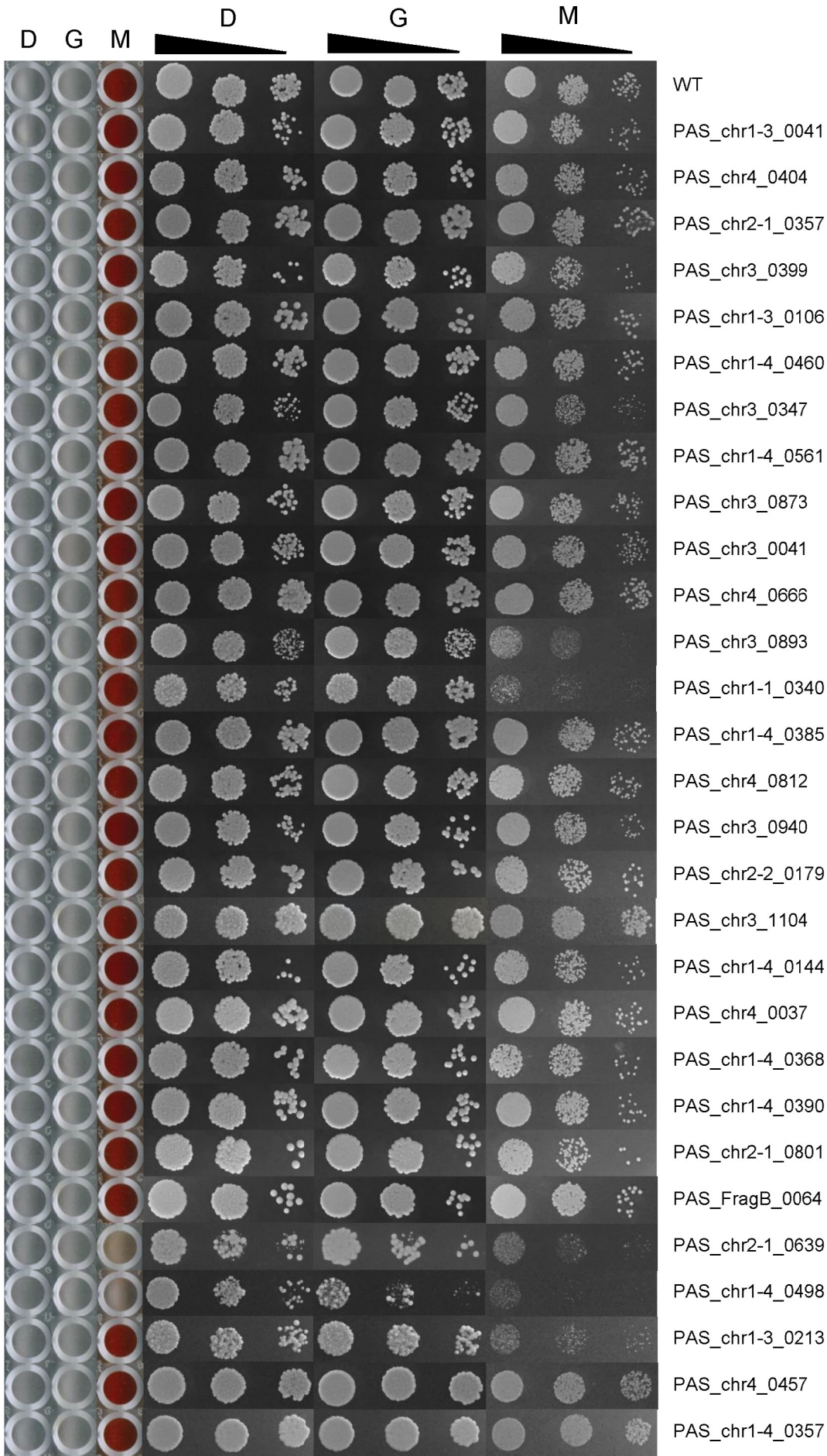

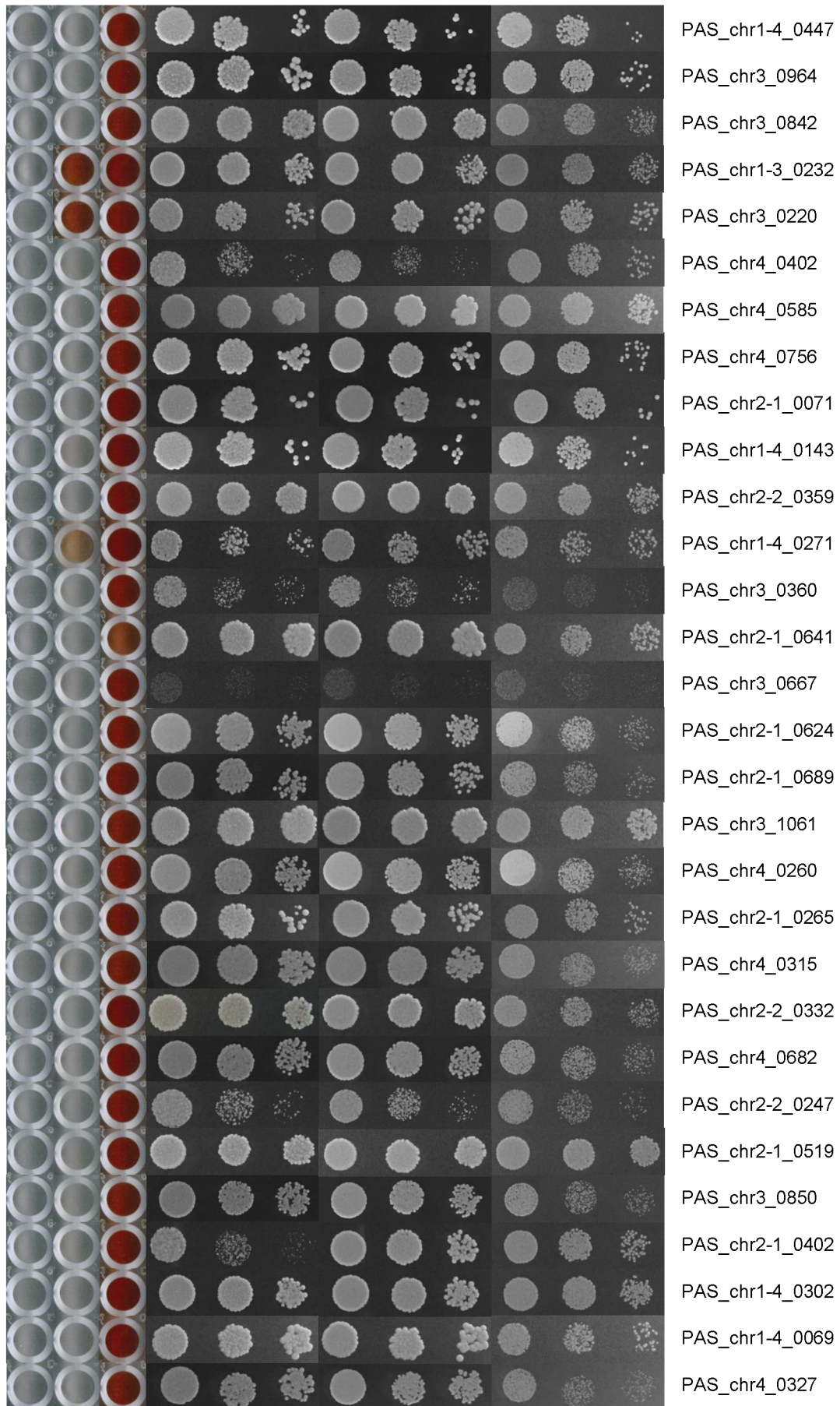

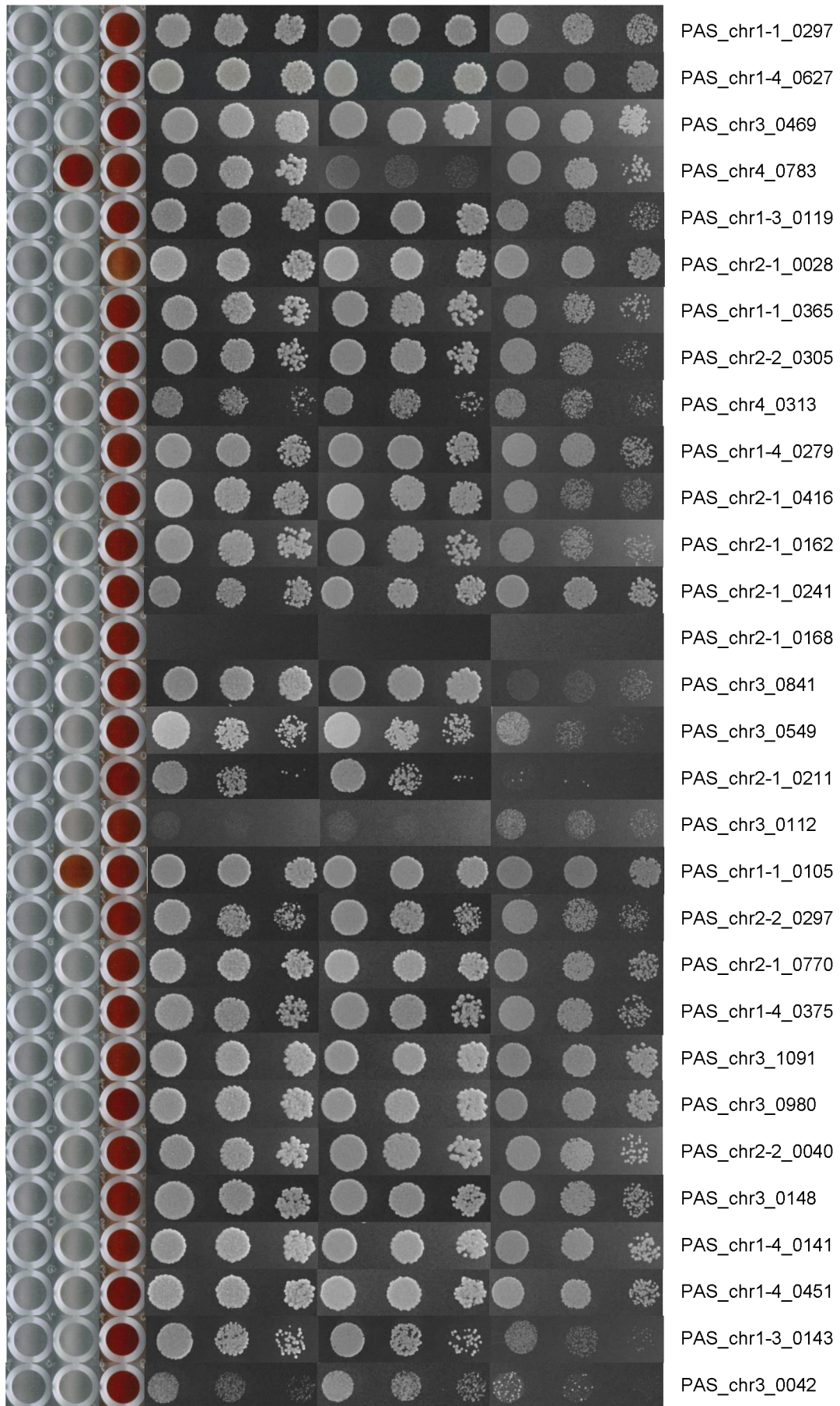

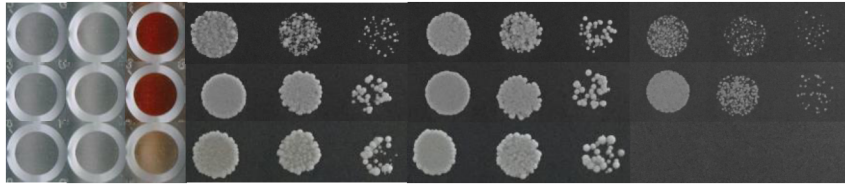

PAS\_chr3\_0072

PAS\_chr2-2\_0103

PAS\_FragB\_0061

Fig B

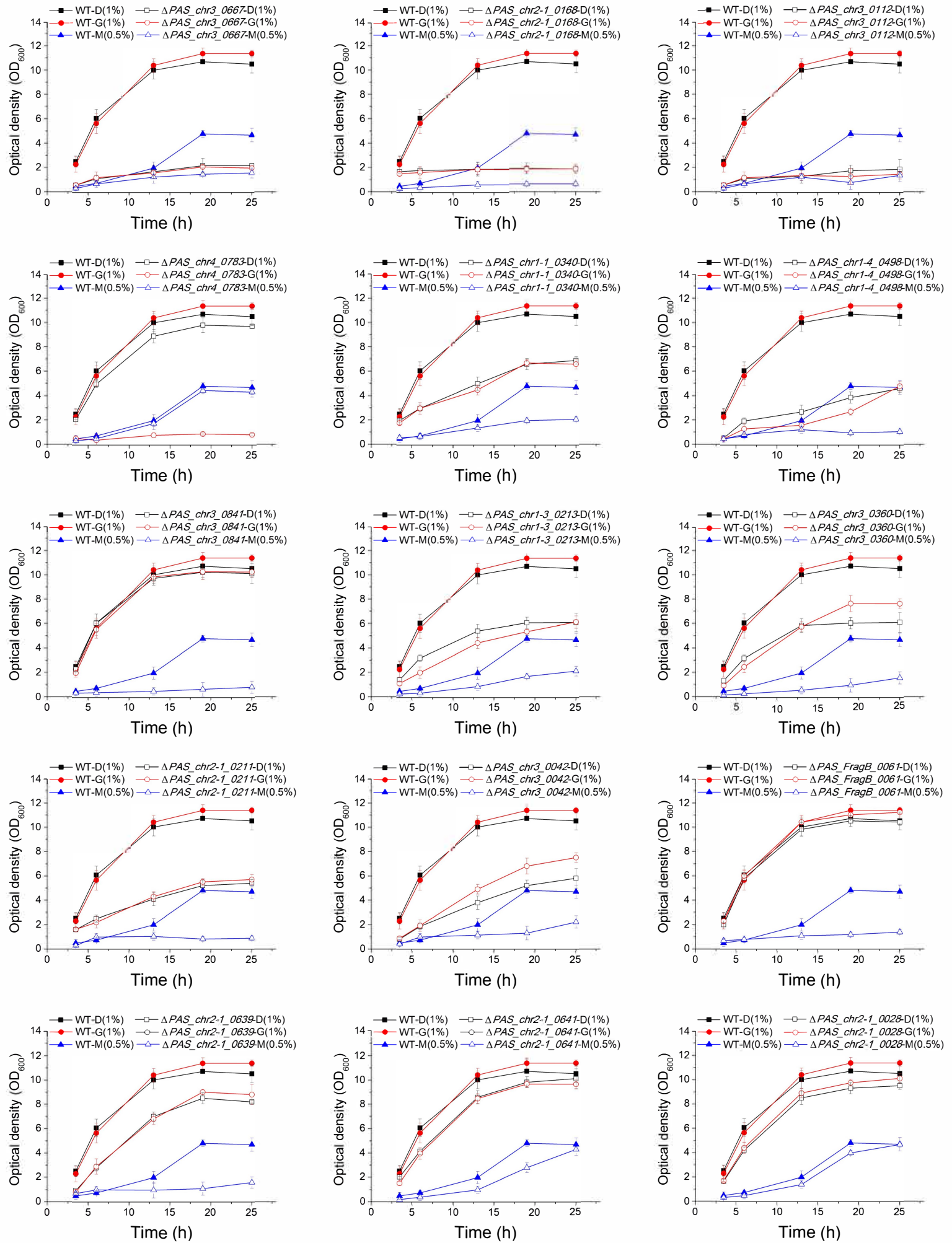

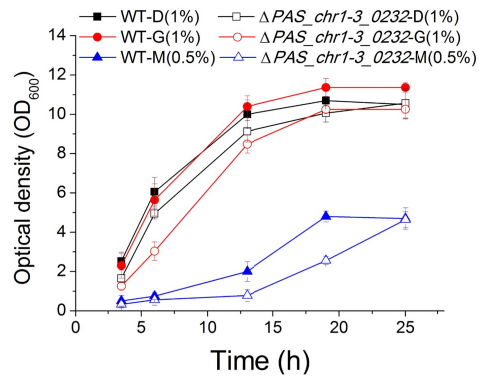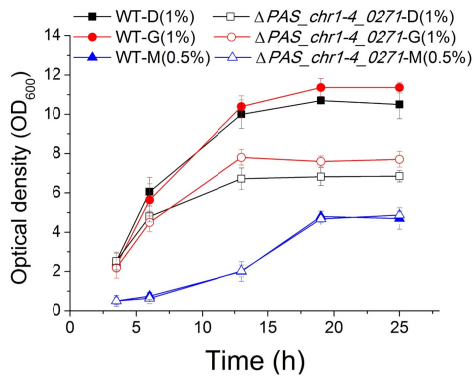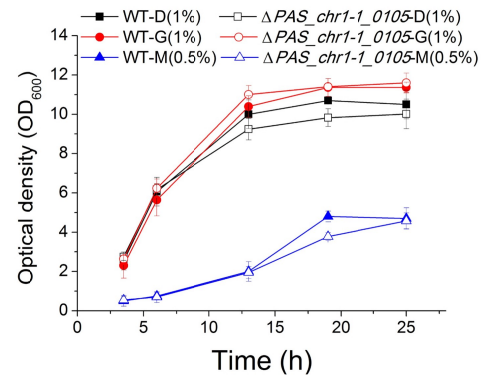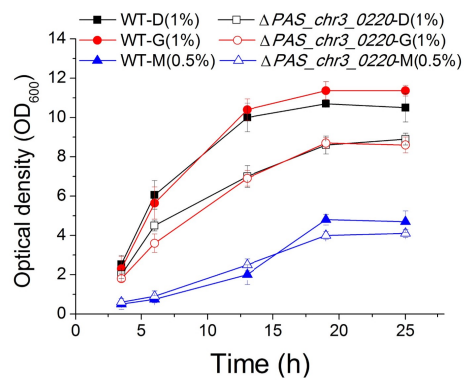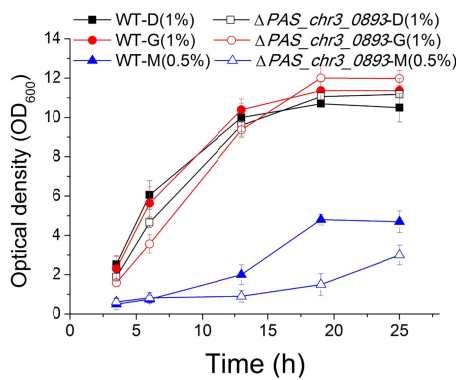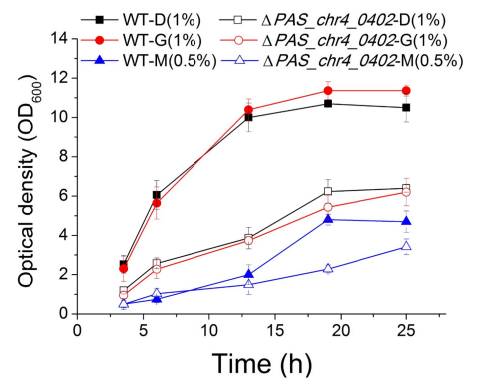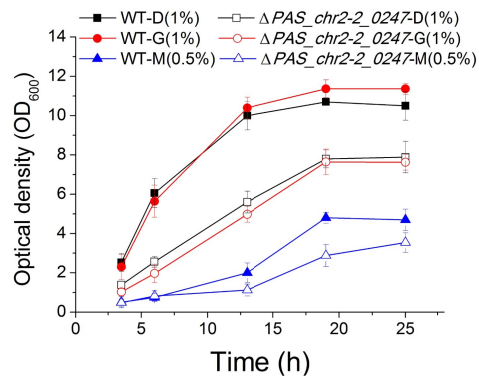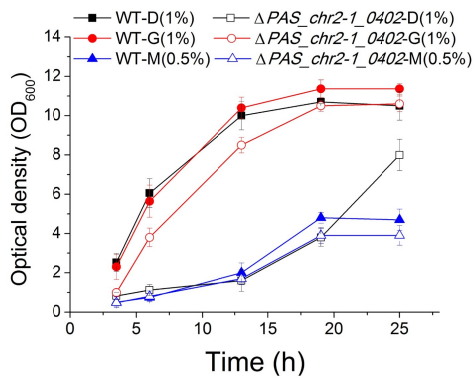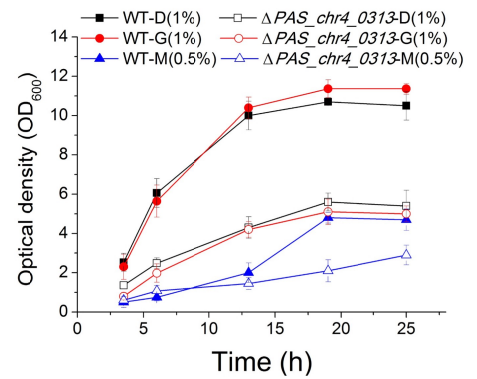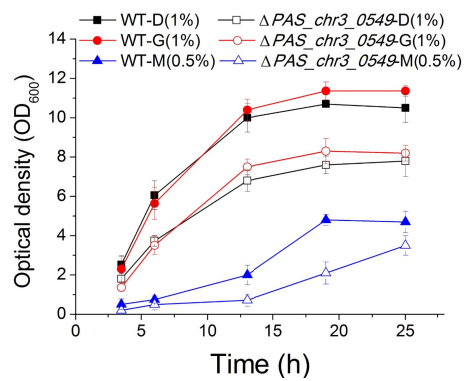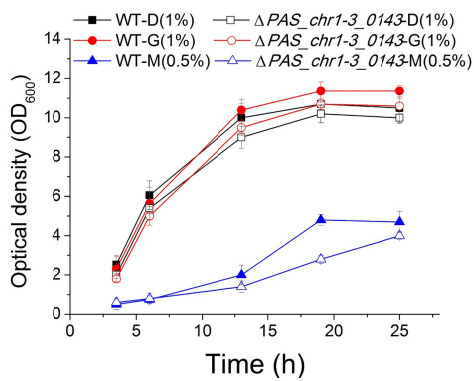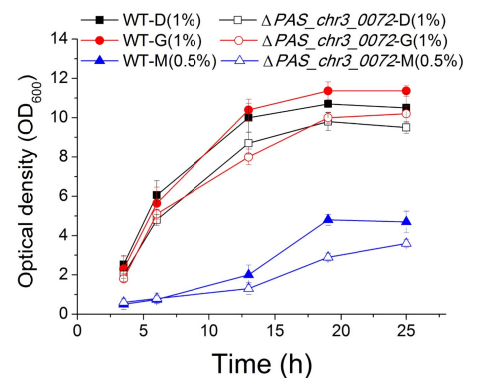

Fig C

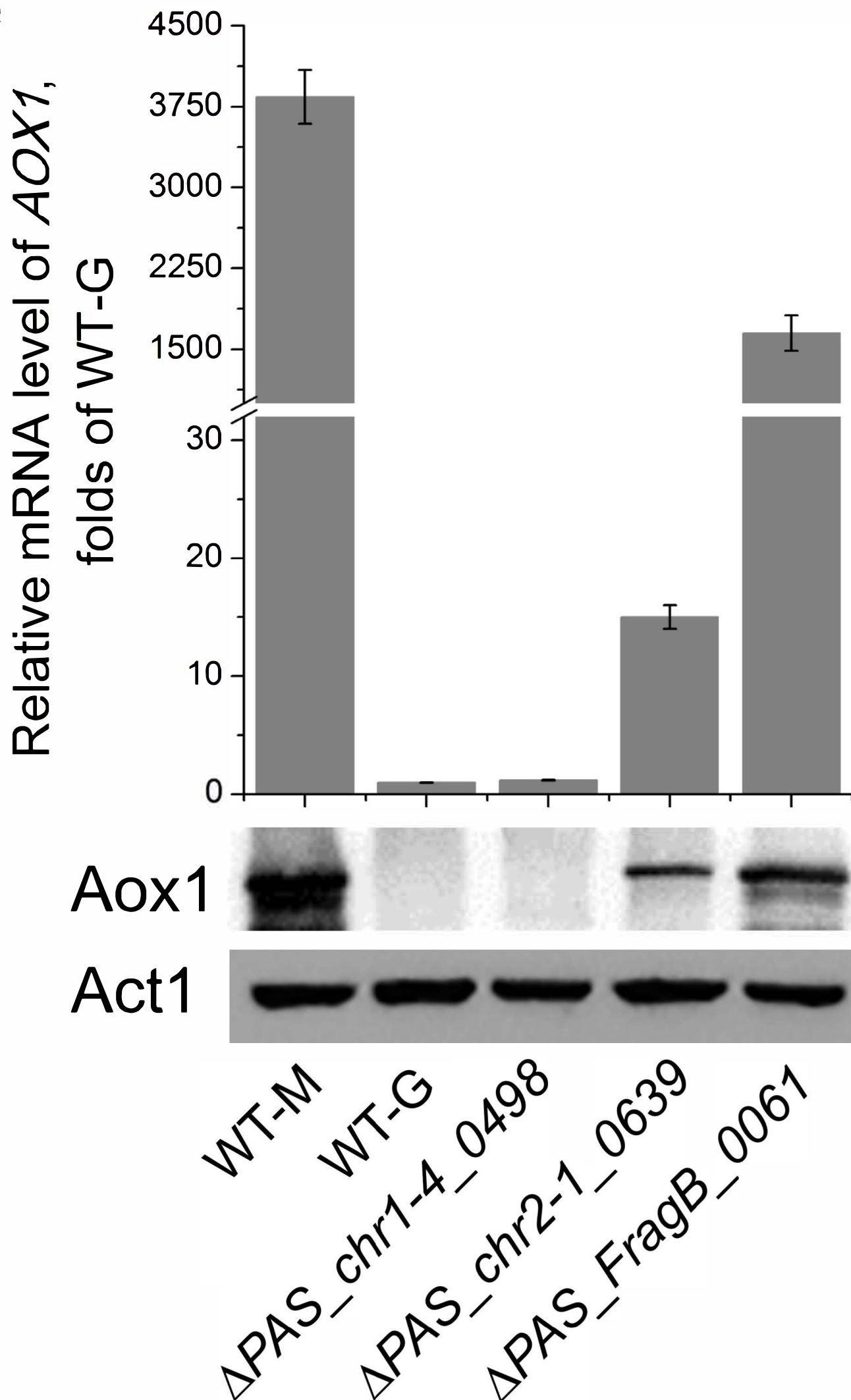

Fig D

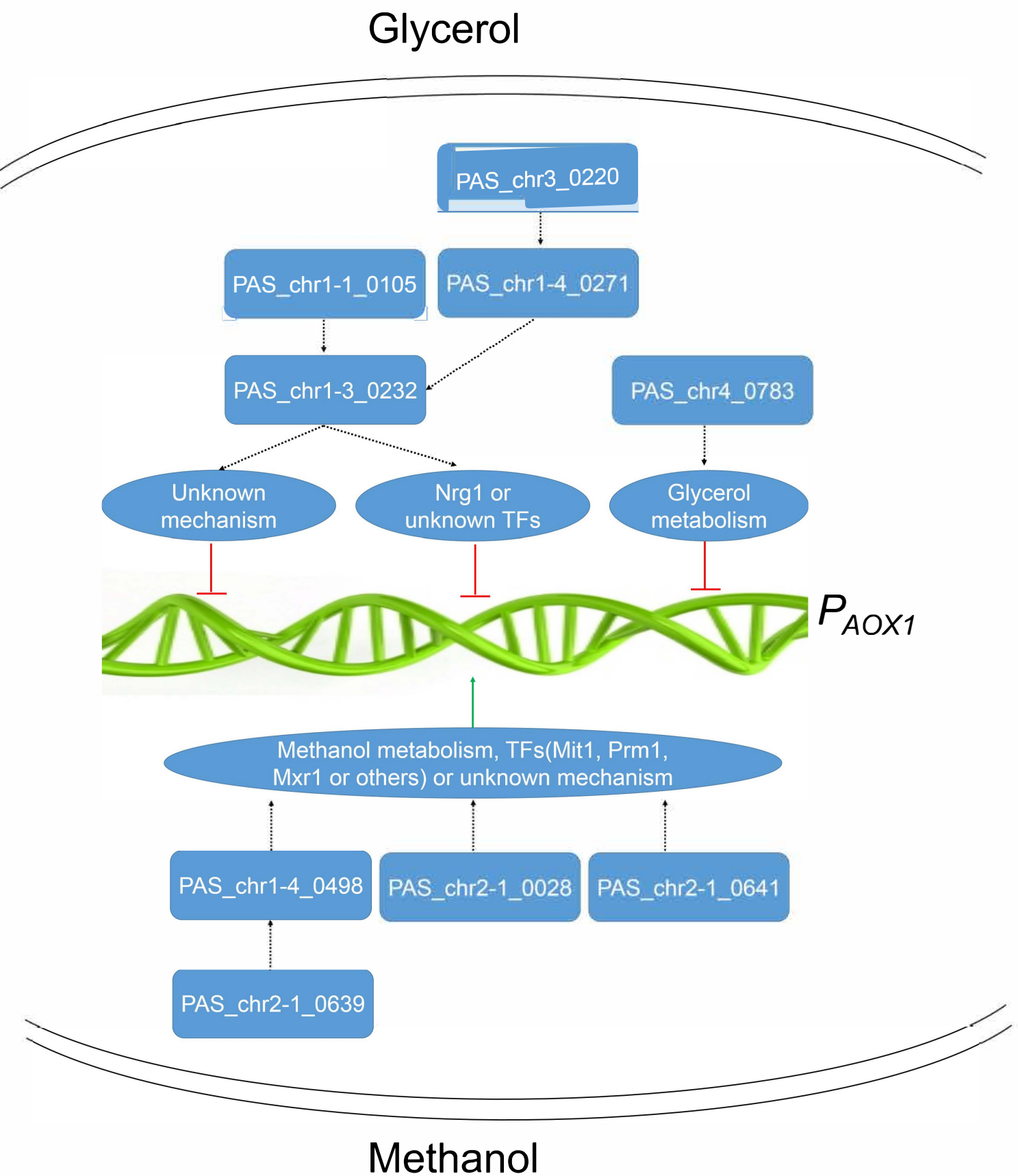

Supplement: S1 File — Fig A: The growth rates (shown by spotting assay) and Aox enzymatic activities (shown by colorimetrical assay) of the 92 knockouts. Fig B: The growth rates (shown by OD measurement of liquid culture) of the 27 affected knockouts. D: glucose; G: glycerol; M: methanol. Fig C: The Aox protein and mRNA levels of three knockouts (ΔPAS_1–4_0498, ΔPAS_FragB_0061 and ΔPAS_chr2-1_0639) cultured in methanol. Methanol and glycerol cultured WT strains (WT-M and WT-G) serve as positive and negative controls, respectively. Actin is blotted as a loading control. Fig D: Putative regulatory pathway of AOX1 promoter in P. pastoris. The regulatory profiles of AOX1 promoter in glycerol and methanol are described. (PDF) [file pone.0167766.s001.pdf]
